# Gender-Related Differences in Mechanics of the Sprint Start and Sprint Acceleration of Top National-Level Sprinters

**DOI:** 10.3390/ijerph17186447

**Published:** 2020-09-04

**Authors:** Dragan M. Mirkov, Olivera M. Knezevic, Amador Garcia-Ramos, Milan Čoh, Nejc Šarabon

**Affiliations:** 1 Faculty of Sport and Physical Education, University of Belgrade, 11000 Belgrade, Serbia; dragan.mirkov@fsfv.bg.ac.rs; 2 Institute for Medical Research, University of Belgrade, 11000 Belgrade, Serbia; olivera.knezevic@imi.bg.ac.rs; 3 Faculty of Sport Sciences, University of Granada, 4090541 Granada, Spain; Amagr@ugr.es; 4 Faculty of Education, Universidad Católica de la Santísima Concepción, 4030000 Concepción, Chile; 5 Faculty of Sport, Ljubljana, University of Ljubljana, 1000 Ljubljana, Slovenia; milan.coh@fsp.uni-lj.si; 6 Faculty of Health Sciences, University of Primorska, 6310 Izola, Slovenia; 7 InnoRenew CoE, Livade 6, 6310 Izola, Slovenia; 8 Andrej Marušič Institute, University of Primorska, 6000 Koper, Slovenia; 9 S2P, Science to practice, Ltd., Laboratory for Motor Control and Motor Behaviour, 1000 Ljubljana, Slovenia

**Keywords:** force–velocity relationship, force platform, kinematics, kinetics, track-and-field

## Abstract

(1) Background: Within the current study we aimed at exploring gender-related differences and the relationship between sprint start block kinematics and kinetics and sprint acceleration force–velocity (F-v) relationship parameters (maximal force [F0], maximal velocity [v0], maximal power [Pmax] and slope) in top national-level sprinters. (2) Methods: Twenty-eight sprinters (6 females) performed 10 maximal 30-m sprints. Start block and acceleration kinematics and kinetics were collected with an instrumented sprint start block and a laser distance sensor (KiSprint system). Displacement-time data were used to determine the F-v relationship through Samozino’s method. (3) Results: Start block rear foot maximal force (effect size [ES] = 1.08), rate of force development (ES = 0.90–1.33), F_0_ (ES = 1.38), v_0_ (ES = 1.83) and P_max_ (ES = 1.95) were higher in males than in females (*p* ≤ 0.05). There were no differences in the slope, and ratio of horizontal-to-resultant force. F_0_, v_0_, and P_max_ generally presented higher correlations with the start block kinetics (median r [range] = 0.49 [0.28, 0.78]) than with the kinematics (median r [range] = −0.27 [−0.52, 0.28]). (4) Conclusions: We confirmed that sprint block phase and sprint acceleration mechanics should be mutually assessed when analyzing sprinting performance. KiSprint system could provide more accurate information regarding mechanical pattern and technique during sprint initiation and acceleration, and potentially help create a more personalized and effective training program.

## 1. Introduction

The ability to quickly accelerate from a static position and maintain high running velocities are clear determinants of performance in sprint race events and also key performance indicators in many other sport disciplines [1,2]. Although numerous studies have been published addressing neurological, physiological and biomechanical determinants of sprinting [3,4,5,6], due to its complexity and myriad of factors affecting sprinting performance, the sprint start continues to attract scientific attention.

Due to the apparently strong influence on total running time, particularly in short distance events (i.e., 60 and 100 m), sprinting block phase performance has been extensively investigated for decades [1,7,8,9]. Since a number of kinematic and kinetic variables have been used to evaluate sprinting block phase performance, the conclusions of those studies were frequently affected by the variable considered (see Bezodis for details; [1]). Another problem with the sprinting literature is that the block start performance has been generally evaluated through non-standardized custom build laboratory settings [8,10,11,12,13], not allowing the direct comparison of mechanical variables among different studies. Nevertheless, most of these studies agreed that attaining a high horizontal power during the block clearance and the ability to quickly accelerate from block clearance are both critical to optimize sprinting performance [8,10,13,14].

After block clearance, achieving and maintaining the maximal horizontal velocity are both related to the sprinter’s capability to apply high amounts of power output, which depends on the capacity to produce high external forces at different running velocities [15,16]. The mutual dependency among force, velocity, and power producing capacities of leg muscles could be well described using the force-velocity (F-v) and power–velocity (P-v) relationships [15,16,17,18,19,20]. However, until recently, the kinetics analysis of the sprint acceleration phase was limited to specialized treadmill ergometers [21] or systems of force plates mounted along the sprint track [22]. Recently, Samozino et al. [16] have proposed a simple and practical method to determine the F-v relationship in running considering only anthropometric and running split-time or velocity-time data. Samozino’s method allows to obtain the sprinter’s maximal power output (P_max_), maximal horizontal force (F_0_), maximal velocity (v_0_), as well as the mechanical effectiveness of the force application onto the ground [15,16,23]. A novel instrumented sprint start block with the laser distance sensor that enables routine collection of the kinematic and kinetic variables during the block phase and kinematic pattern of the sprint acceleration phase has recently appeared on the market (KiSprint system, Kistler Instrumente GmbH, Winterthur, Switzerland). With this technology, coaches and researchers could collect important and more accurate information regarding mechanical pattern and technique during sprint initiation and acceleration daily and deliver a potentially more personalized and effective training program. Further, this would allow better understanding of important features regarding the biomechanics of sprint block and acceleration phase and their potential relationship.

Various factors may affect the magnitude and associations of the variables collected in the block and acceleration phases of the sprint. In addition to age, level of performance, and technique, gender is a well-recognized factor that influences performance during both phases [1,24]. Few studies have investigated the gender-related differences in the kinematic and kinetic performance indices of the sprint start [1,10]. The most prominent differences were detected for the kinetic variables with males exhibiting higher maximal and average force production, as well as higher force impulse values [10]. Gender-related differences in F-v relationship parameters have also been reported with males exhibiting generally higher F_0_ and P_max_ than their female counterparts [24]. However, although these results are evident when the absolute values of force and velocity are considered, contrasting findings may exist when force and power values are normalized to body mass. Although seminal work of Jaric et al. [25] has proven that muscle strength and power are not linearly proportional to body mass, suggesting the allometric scaling as a method for normalization, particularly when comparing different populations, no previous study has compared allometrically scaled F-v relationship parameters between male and female sprinters. Only recently, Slavinski et al. [24] have compared the F-v relationships obtained during an official race between female and male world-class sprinters showing that all of the mechanical variables normalized to body mass were greater for males. Although this study yields important findings regarding the gender-related differences in mechanical output variables related to the sprint acceleration phase, data from the start block were not included.

Having all these in mind, the present study compared (a) start block kinematics and kinetics and (b) F-v relationship parameters obtained from the sprint acceleration between young top national-level male and female sprinters. In addition, we aimed to explore the relationship between sprint block kinetic and kinematics and F-v relationship parameters obtained during the sprint acceleration phase. We hypothesized that (a) despite allometric scaling, male athletes would have better sprint start and acceleration mechanics than females, and (b) F_0_, v_0_, and P_max_ would present higher association with the start block kinetics than with kinematics. We expect that this study will contribute to a better understanding of gender-related differences in muscle mechanical capacities, as well as their role in sprint block clearance and acceleration.

## 2. Materials and Methods

### 2.1. Experimental Design

A cross-sectional study was conducted to compare mechanical performance during the block and acceleration phases of the sprint between top national-level male and female sprinters. Subjects were tested in a single session that consisted of 10 maximal sprints of 30 m. To control for possible effects of body size, all kinetic variables from sprint start block and F-v relationship parameters were normalized to body mass using an allometric scaling approach [25]. All sprinters were tested between 9 a.m. and 5 p.m. and under similar environmental conditions (~25 °C and ~50% relative humidity).

### 2.2. Subjects

Twenty-two male (age: 17.7 ± 2.7 (16–24) years; body mass: 74.7 ± 7.8 kg; height: 1.81 ± 0.06 m) and six female (age: 17.0 ± 2.0 (15–24) years; body mass: 58.7 ± 5.8 kg; height: 1.66 ± 0.03 m) top national-level from Slovenia were selected to participate in the study. Subjects were asked to refrain from intense physical activities at least 24 h prior to testing. At the time when the study was performed, all subjects were healthy with no reported injuries in last two years. All subjects and their parents/guardians were informed in detail about the study procedure and thereafter gave their written consent to participate in this study, which was approved by the National Medical Ethics Committee in agreement with the Declaration of Helsinki.

### 2.3. Testing Procedures

After a general warm-up (10 min of low intensity running, 8 repetitions of dynamic stretching exercises for the main muscle groups, 10 repetitions of squats, push-ups and crunches), the subjects performed specific sprint drills for 10–15 min at their individual choice. The warm-up was led by a track-and-field coach. After that, the sprinters were given time (~5 min) to position the start blocks for their best fit and to perform 2–3 submaximal familiarization trials. The experimental session consisted of 10 maximal 30-m sprints separated by 3 min of rest. The arithmetic mean value of the 10 repetitions was used for statistical analyses.

**Figure 1:**
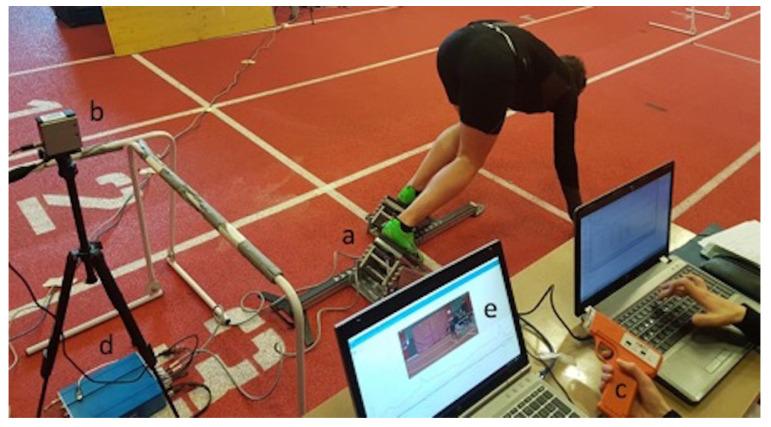
Measurement set-up: 3-dimensional force measuring start blocks (**a**), laser distance sensor on a tripod (**b**) and start signal pistol (**c**), connected via the amplifier and data acquisition box (**d**) to the computer for storage and off-line analysis (**e**).

Kinematic and kinetic variables during the block phase were collected with an instrumented sprint start block (KiSprint system, Kistler Instrumente GmbH, Winterthur, Switzerland), a part of which is also the laser distance sensor used for the 5-, 10-, 20-, and 30-m split times measurements (for details see Figure 1). The following kinematic sprint start variables were analyzed: (1) reaction time (ms)—the time interval from the start gun signal to the moment when the common (front + rear foot) force reaches the threshold of 200 N above the baseline during the “set” position; (2) block time (ms)—time interval between the start gun signal and take off defined as the moment when the common force drops to 0; (3) horizontal take-off velocity (m·s^−1^)—horizontal velocity at the moment of the take-off calculated from the force impulse; and (4) rear and front leg push-off time (ms)—time from each leg’s start of the push-off to the moment when the sagittal plane common force of the related block drops to 0. The following kinetic sprint start variables were analyzed: (1) average horizontal power (W·kg^−2/3^); (2) maximal force of the rear and front leg (N·kg^−2/3^)—peak sagittal plane common force produced by the rear and front leg during the push-off; and (3) maximal and average rate of force development (RFD; N × s^−1^·kg^−2/3^) of the rear and front leg—the peak of the sagittal plane common force time derivative and the mean of the RFD during the ascending phase of common force during the push-off action, respectively. A simple biomechanical model recently proposed by Samozino et al. [16] was applied to obtain the F-v relationship. This method is based on a macroscopic biomechanical model, which requires only anthropometric (body mass and height) and spatio-temporal (split times) input variables. F-v relationships where derived for each runner and extrapolated to obtain F_0_, v_0_, and the slope of the F-v relationship, while Pmax was calculated as Pmax = (F_0_ × v_0_)/4. The mechanical effectiveness of force application during running (DRF; decrease in the ratio of horizontal-to-resultant force) was also assessed as the slope of RF-v relationship, where RF presents the ratio of horizontal force and the corresponding resultant ground reaction force (i.e., index of force application technique) [19].

### 2.4. Statistical Analyses

Descriptive data of the dependent variables are presented as means and standard deviations. Normal distribution of the data was confirmed by the Shapiro–Wilk test (*p* > 0.05). Gender-related differences in dependent variables were assessed through independent samples t-tests, which has been proven to be highly robust with respect to (a) small; (b) unequal sample size [26]. In addition, to adequately interpret gender related differences and the Cohen’s d effect size (ES). The magnitude of the ES was quantified using the following scale: negligible (<0.20), small (0.20–0.49), moderate (0.50–0.79), and large (≥0.80). Relationship between kinematic and kinetic sprint block metrics and F-v relationship parameters was estimated through Pearson’s correlation coefficients (r). Qualitative interpretations of the r coefficients (trivial [0.00–0.09], small [0.10–0.29], moderate [0.30–0.49], large [0.50–0.69], very large [0.70–0.89], nearly perfect [0.90–0.99], and perfect [1.00]) [27] are provided for all significant correlations. All statistical analyses were performed using the SPSS (IBM SPSS version 25.0, Chicago, IL, USA) software package with statistical significance set at an alpha level of 0.05 (two-tailed).

## 3. Results

**Figure 2:**
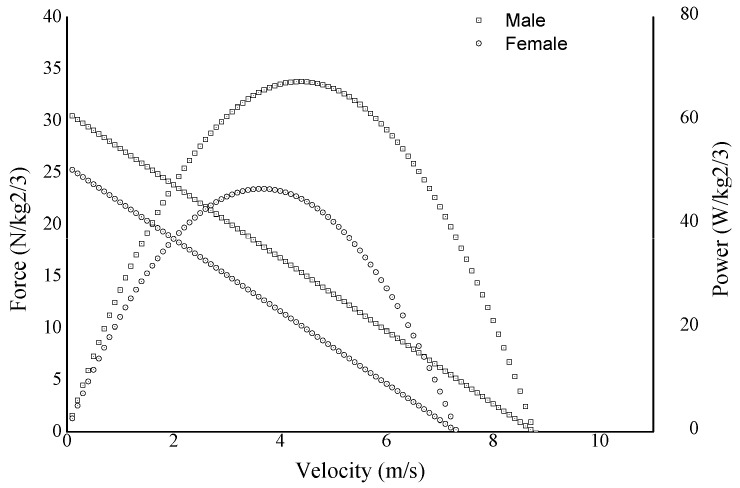
Averaged across the subjects’ force– and power–velocity relationship profiles obtained from the sprint acceleration separately for males and females.

Descriptive statistics for kinematic and kinetic sprint start variables obtained from sensors positioned in the start blocks as well as for the parameters derived from the F-v relationships are presented in Table 1 and Table 2. Graphical representation of F-v and P-v relationships averaged across subjects, separately for males and females, are available in Figure 2.

### 3.1. Gender-Related Differences

The comparison of kinematic and kinetic sprint–start variables between top national-level male and female sprinters generally evidenced a better block start performance for men. Specifically, five variables showed a large effect (push-off time of the front leg, maximal force of the rear leg, maximal and average RFD of the rear leg, and horizontal maximal power), two variables a moderate effect (push-off time of the rear leg and horizonal velocity), four variables a small effect (block time, maximal force of the front leg, and maximal and average RFD of the front leg), and only one variable a negligible effect (reaction time). Regarding the comparison of the parameters derived from the F-v relationship, large effects in favor of males were observed for three variables (F_0_, v_0_, and P_max_) but negligible differences were observed for the slope of the F-v relationship and D_RF_.

### 3.2. Relationship between the Variables Collected in the Block and Acceleration Phases

Pearson’s correlation coefficients between kinematic and kinetic sprint block metrics and F-v relationship parameters are presented in Table 3. The relationship between kinematic sprint block metrics and F-v relationship parameters was generally negative ranging from low to moderate with the highest correlation being observed between the push-off time of the front leg and F_0_ and P_max_. Regarding the relationship between the kinetic sprint block metrics and F-v relationship parameters, positive moderate to very large correlations were observed between F_0_ and P_max_ with maximum force of the front leg, average RFD of the front leg, and maximal horizontal power.

## 4. Discussion

This study was designed to compare the kinematic and kinetic variables collected during the block phase, and F-v relationship parameters obtained during the acceleration phase between top national-level male and female track and field athletes. In addition, the relationship between the sprint block kinetics and kinematics and the F-v relationship parameters was investigated. To our knowledge, this is the first study that explored gender-related differences on allometrically scaled mechanics of both sprint start and sprint acceleration. The main findings are that top national-level male sprinters compared to female sprinters exhibited greater start block rear foot maximum force and rate of force development, along with higher force, velocity, and power producing capacities during the sprint acceleration. Moreover, it could be of importance that slope of the F-v relationship and the mechanical effectiveness of force application during running were similar for males and females. Finally, only start block kinetics were positively associated with the mechanical capacities of muscles responsible for acceleration during sprint running.

### 4.1. Gender-Related Differences in Start Block Kinematics and Kinetics

The sprinters’ reaction time has been widely investigated [1,3,5,7], and the studies, in contrast to our findings, generally reported shorter reaction times for males compared to females [7,28]. However, it has been shown that reaction time presents a high between- and within-subjects variability, and many factors such as block technique [29], the sprinter’s focus of attention [30], start signal type [1], measurement methodology, and performance level [28] could all affect the reaction time, making it difficult for interpretation. In addition to reaction time, the push-off phase duration has also been recognized as an important contributor to the success in sprint races with a shorter time spent on the block being associated with a better sprint performance [1,29]. In the current study, results of both male and female athletes are in line with the values reported in the literature Čoh et al. [8,13]. In addition, the shorter push-off times as well as higher horizontal push-off velocities in males are consistent with the finding of Čoh et al. [8]. Nevertheless, a lack of more research data as well as the complex relationship between horizontal push-off velocity and different segmental kinematic and kinetic variables do not allow a more in-depth interpretation of the obtained findings [1].

In line with our first hypothesis, larger between-gender differences were recorded for the rear leg push-off variables in comparison to the front leg, with males exerting higher forces with the rear foot and particularly higher rate of force development. Consequently, albeit not significant, the resulting horizontal power was also higher for males. Since maximal horizonal push-off velocity and power are positively related to a better sprint outcome, this is one of the factors that explains why male athletes are generally achieving better results in sprint than female athletes [24].

### 4.2. Gender-Related Differences in F-V Relationship Parameters

Regarding the kinetic variables, both F-v and P-v profiles clearly differed between males and females with males showing higher force (F_0_), velocity (v_0_) and power (P_max_) producing capacities. These findings are in line with those published in a recent study aimed to compare the F-V relationship parameters of female and male world-class sprinters [24]. Similar findings have also been observed in studies reporting the F-V relationship parameters in cycling [31]. However, most studies addressing gender-related differences failed to report data relative to body mass or reported data applying a scaling ratio, despite the well-known physiological principle that muscle force and power producing capacities do not raise linearly with the increase in body mass [25]. This compromises the comparison of results among different studies. To eliminate the confounding effect of body size, F-v relationship parameters within the current study were allometrically scaled relative to body mass. Just as hypothesized, even after normalization, all F_0_, v_0_, and P_max_ were higher in male sprinters, suggesting that other factors besides body mass should be responsible for the higher muscular capacities that are observed in males. Namely, studies exploring sprint performance have shown that stride length rather than stride frequency is responsible for higher sprint achievement [32,33], implicating that faster runners produce higher ground reaction forces (and thus higher horizontal forces) [32], allowing them to achieve higher velocities [32,33]. Besides the greater muscle fiber cross-sectional area in males, higher capacities to produce force in males could be attributed to higher concentration of circulating testosterone and its effects on skeletal muscle contraction capacities [33]. Nevertheless, the role of factors other than body size contributing to higher F-v relationship parameters in males than in females should be further investigated. Finally, our study showed that after allometric scaling, the slope of the F-v relationship, and the mechanical effectiveness of force application during running were similar for males and females. Since mechanical effectiveness of force application during running represents how effectively the total force developed by the lower limbs is applied onto the ground, even with increasing speed during the acceleration phase (i.e., index of force application technique) [19], our findings indicate that despite lower force and power-producing capacities, female sprint runners could achieve equal technical ability of force application as their male counterparts. This implies that both F-v relationship parameters in sprint and mechanical effectiveness of force application during running should be used in standard assessment of sprinters’ performance.

### 4.3. Relationship between F-V Relationship Parameters in Sprint and Start Block Kinematics and Kinetics

Among the kinematic variables from the start block phase, only the front leg push-off time was moderately correlated to F_0_ and P_max_. Previous studies have shown that among other factors, optimal performance in sprinting is related to the sprinter’s ability to produce higher horizontal impulses [1,7], which could be maximized through achieving higher force, increasing the duration of the push off phase, or both [1,12]. Since the contribution of the front leg to the total horizontal impulse is around 66–76% [1,10,13] due to 1.9–2.4 times longer block contact than the rear leg [34], achieving longer front leg times could be positively related to the efficacy of the lower leg muscles mechanical capacity.

Regarding our second hypothesis, moderate to the strong relationships between lower limb start block dynamics and F-v relationship parameters from sprint acceleration, were generally obtained. The associations of F0 and Pmax were generally higher with front leg start block dynamics (maximal force and rate of force development) than with rear leg start block dynamics. The average horizontal start block power was also strongly correlated to F_0_ and P_max_. This is in line with findings from studies describing the factors underlying effective transition from sprint block clearance to sprint running [1,8,10]. Namely, it is well known that achieving optimal sprint performance depends on the sprinters’ ability to reach the maximum horizontal power during block clearance and further accelerating during the transition phase from block clearance into sprint running [1,10]. On the other side, it has been shown that the ability to exert a large propulsive force [35] as well the orientation of the total force applied onto the supporting ground during sprint acceleration [15] are essential for achieving greater acceleration and maintaining higher maximal speed.

Our study confirms that sprint block phase and sprint acceleration mechanics should be mutually assessed when analyzing sprinting performance. For that matter, the KiSprint, which consists of an instrumented sprint start block and a laser distance sensor, allows routine monitoring of sprint training. Thus, practitioners could use the same device to evaluate both the block start performance and the F-v relationship parameters during the acceleration phase. This procedure has been shown effective to discriminate between high-level male and female track and field sprinters. Furthermore, coaches could collect important information daily, regarding not only the important mechanical pattern but also technique during sprint initiation and acceleration. Finally, this would allow deeper exploration of important features regarding the biomechanics of sprint block and acceleration and their potential relationship.

The main limitation of the current study is the relative heterogeneity of the study sample. Due to the small number of elite senior sprinters, particularly females, elite junior sprinters were included in the study, increasing the variability in the start block dynamics, particularly when assessing the rate of force development. However, note that study sample comprised almost all national level short-distance runners. Nevertheless, we have limited our conclusions to the population explored, while future studies, performed on larger sample size, should be performed to confirm generalization of our findings. Another limitation could be the relatively short distance of the acceleration phase. Most studies have reported the F-v relationship parameters during linear sprints of 40 m. Nevertheless, a recent study that aimed to explore the F-v profile of female soccer players has shown that a sprint of 30 m could be used to extract the mechanical capacities since the maximum speed was reached before reaching a distance of 30 m [2,36]. Finally, although body size presents an important contributing factor when observing differences in mechanical capacities, other, particularly physiological factors should be considered when interpreting the findings obtained from the current study on male and female sprinters.

## 5. Conclusions

The main findings of the study are that even after force and power values were allometrically scaled relative to body mass male sprinters exhibited a better block start performance (i.e., greater rear foot maximum and explosive strength) and sprint acceleration performance (i.e., greater values of F0, v0, and Pmax) compared to females. However, despite lower force and power-producing capacities, female sprint runners could achieve equal technical ability of force application during the acceleration phase as their male counterparts. Only start block kinetics were related to the force and power producing capacities of muscles responsible for acceleration during sprint running. Nevertheless, our findings imply that that sprint block phase and sprint acceleration mechanics should be mutually assessed when analyzing sprinting performance. In addition, they contribute to a better understanding of gender-related differences, as well as the role of muscle capacities in sprint block clearance and acceleration. This could be of importance not only for the improvement of sprint training of sprinters but also in all sport disciplines were sprint acceleration is important for sport specific performance. Although some of our findings confirm the ones published previously, this is the first study that used the novel start block measurement system integrated with the option to assess the F-V relationship from the acceleration phase of the sprint. Therefore, we were able to provide new insight into mechanical muscle capacities responsible for sprint initiation and acceleration. Finally, from the practical point of view, we have shown that sprint initiation and acceleration could be routinely assessed with KiSprint system, thus providing more accurate information regarding mechanical pattern and technique, and potentially help create a more personalized and effective training program.

## Figures and Tables

**Table 1:** Comparison of kinematic and kinetic sprint start variables between males and females.

Variable	Males	Females	*t* Value	*p* Value	ES
Kinematic Variables					
Reaction time (ms)	134 ± 24	134 ± 23	0.06	0.95	0.03
Push-off time—rear leg (ms)	173 ± 16	184 ± 19	−1.35	0.19	0.62
Push-off time—front leg (ms)	345 ± 34	373 ± 32	−1.78	0.09	0.82
Block time (ms)	483 ± 39	497 ± 41	−0.78	0.44	0.36
Horizontal take-off velocity (m·s^−1^)	3.20 ± 0.30	3.03 ± 0.37	1.16	0.26	0.53
Kinetic Variables					
Maximal force—rear leg (N·kg^−2/3^)	5.94 ± 1.24	4.62 ± 1.03	2.35	0.03	1.08
Maximal force—front leg (N·kg^−2/3^)	7.30 ± 0.88	7.07 ± 0.62	0.57	0.57	0.26
Maximal RFD—rear leg (N × s^−1^·kg^−2/3^)	140 ± 57	92 ± 30	1.96	0.06	0.90
Maximal RFD—front leg (N·s^−1^·kg^−2/3^)	88.9 ± 32.9	76.4 ± 9.6	0.91	0.15	0.42
Average RFD—rear leg (N·s^−1^·kg^−2/3^)	58.1 ± 17.9	36.0 ± 9.3	2.88	0.01	1.33
Average RFD—front leg (N·s^−1^·kg^−2/3^)	24.1 ± 5.4	21.6 ± 4.1	1.02	0.32	0.47
Average horizontal power (W·kg^−2/3^)	59 ± 14	48 ± 15	1.77	0.09	0.81

Means ± standard deviation. ES, Cohen’s d effect size. RFD, rate of force development.

**Table 2:** Comparison of the sprint force–velocity relationship parameters between males and females.

Variable	Males	Females	*t* Value	*p* Value	ES
*v*_0_ (m·s^−1^)	8.64 ± 0.46	7.85 ± 0.24	3.93	0.01	1.83
*F*_0_ (N·kg^−2/3^)	30.8 ± 4.1	25.6 ± 1.8	3.01	0.01	1.38
Fv slope (N·s·kg^−2/3^·m^−1^)	−3.52 ± −0.57	−3.50 ± −0.37	−0.07	0.94	0.02
P_max_ (N·kg^−2/3^)	87.7 ± 15.8	59.4 ± 6.7	4.24	0.01	1.95
D_RF_ (%)	−8.01 ± 1.14	−8.03 ± 0.50	0.03	0.98	0.01

ES, Cohen’s d effect size; F_0_, theoretical maximal force; v_0_, theoretical maximal velocity; Fv slope, slope of the force–velocity relationship; P_max_, maximal power; D_RF_, decrease in the ratio of horizontal-to-resultant force.

**Table 3:** Correlations between sprint start block kinematics and kinetics and the force–velocity relationship parameters.

Variable	v_0_	F_0_	P_max_	Fv Slope	D_RF_
Start block kinematics					
Reaction time	−0.04	−0.3	−0.27	0.32	0.37
Push-off time—rear leg	−0.23	−0.28	−0.32	0.22	0.04
Push-off time—front leg	−0.26	−0.51 **	−0.52 **	0.46 *	0.33
Block time	−0.16	−0.38 *	−0.38 *	0.35	0.40 *
Horizontal take-off velocity	0.05	0.28	0.24	−0.3	−0.34
Start block kinetics					
Maximal RFD—rear leg	0.32	0.28	0.32	−0.19	0.13
Maximal RFD—front leg	0.31	0.51 **	0.53 **	−0.50 *	−0.01
Average RFD—rear leg	0.45 *	0.45 *	0.52 **	−0.34	0.09
Average RFD—front leg	0.40 *	0.70 **	0.72 **	−0.63 **	−0.32
Maximal force—rear leg	0.49 *	0.48 *	0.55 **	−0.36	0.01
Maximal force—front leg	0.48 *	0.75 **	0.78 **	−0.65 **	−0.29
Average horizontal power	0.32	0.70 **	0.70 **	−0.66 **	−0.39 *

RFD, rate of force development; F_0_, theoretical maximal force; v0, theoretical maximal velocity; F-v slope, slope of the force–velocity relationship; P_max_, maximal power; D_RF_, decrease in the ratio of horizontal-to-resultant force. *, *p* < 0.05; **, *p* < 0.01.
